# Impact of technology-based interventions for children and young people with type 1 diabetes on key diabetes self-management behaviours and prerequisites: a systematic review

**DOI:** 10.1186/s12902-018-0331-6

**Published:** 2019-01-10

**Authors:** Emily C. L. Knox, Helen Quirk, Cris Glazebrook, Tabitha Randell, Holly Blake

**Affiliations:** 1 University of Nottingham, School of Health Sciences, Nottingham, UK; 2 Sheffield Hallam University, Centre for Sport and Exercise Science, Sheffield, UK; 3 University of Nottingham, School of Medicine, Nottingham, UK; 4 Department of Paediatric Endocrinology and Diabetes, Nottingham Children’s Hospital, Nottingham University Hospitals NHS Trust, Nottingham, UK; 5 NIHR Nottingham Biomedical Research Centre, Nottingham, UK

**Keywords:** Type one diabetes mellitus, Child and young people, Health, Technology, Intervention, Self-management

## Abstract

**Background:**

The role of technology in the self-management of type 1 diabetes mellitus (T1DM) among children and young people is not well understood. Interventions should aim to improve key diabetes self-management behaviours (self-management of blood glucose, insulin administration, physical activity and dietary behaviours) and prerequisites (psychological outcomes and HbA1c) highlighted in the UK guidelines of the National Institute for Health and Care Excellence (NICE) for management of T1DM. The purpose was to identify evidence to assess the effectiveness of technological tools in promoting aspects of these guidelines amongst children and young people.

**Methods:**

A systematic review of English language articles was conducted using the following databases: Web of Science, PubMed, Scopus, NUSearch, SAGE Journals, SpringerLink, Google Scholar, Science Direct, Sport Discus, Embase, Psychinfo and Cochrane Trials. Search terms included paediatric, type one diabetes, technology, intervention and various synonyms. Included studies examined interventions which supplemented usual care with a health care strategy primarily delivered through a technology-based medium (e.g. mobile phone, website, activity monitor) with the aim of engaging children and young people with T1DM directly in their diabetes healthcare. Studies did not need to include a comparator condition and could be randomised, non-randomised or cohort studies but not single-case studies.

**Results:**

Of 30 included studies (21 RCTs), the majority measured self-monitoring of blood glucose monitoring (SMBG) frequency, clinical indicators of diabetes self-management (e.g. HbA1c) and/or psychological or cognitive outcomes. The most positive findings were associated with technology-based health interventions targeting SMBG as a behavioural outcome, with some benefits found for clinical and/or psychological diabetes self-management outcomes. Technological interventions were well accepted by children and young people. For the majority of included outcomes, clinical relevance was deemed to be little or none.

**Conclusions:**

More research is required to assess which elements of interventions are most likely to produce beneficial behavioural outcomes. To produce clinically relevant outcomes, interventions may need to be delivered for at least 1 year and should consider targeting individuals with poorly managed diabetes. It is not possible to determine the impact of technology-based interventions on insulin administration, dietary habits and/or physical activity behaviour due to lack of evidence.

## Background

Type 1 diabetes mellitus (T1DM) is increasing in prevalence in the UK, affecting over 30,000 children and young people and so diabetes management typically begins early in life [1]. The management of T1DM is complex and involves key self-management behaviours outlined in national recommendations (NICE), including: self-monitoring of blood glucose (SMBG), insulin administration, dietary management and regular physical activity, with the aim of maintaining optimal blood glucose levels [2]. Further, NICE guidance highlights key psychological and clinical (HbA1c) prerequisites to self-management behaviour [2]. A focus on any or all of these components will lead to improved diabetes control [2].

Technology-based interventions may augment face-to-face interactions with therapeutic staff, thereby offering the potential to reduce the costs and dependence on clinical staff of providing additional services beyond regular clinical visits. Efficacious delivery of policy objectives in clinical care through technology-based mediums could therefore contribute to reducing the economic burden of T1DM. Technology-based interventions also augment standard services, increasing access and availability of evidence-based practices outside clinical settings. This could be a useful adjunct to the usual care of children and young people with T1DM, particularly as increasing independence and self-reliance is a concern amongst this group [3]. Prior non-systematic reviews [4–6] demonstrate that technology-based interventions promoting diabetes self-management may be acceptable to children and young people with T1DM, and have the potential to improve certain outcomes and behaviours (e.g. HbA1c, blood glucose monitoring).

The purpose of this systematic review is to critically analyse the literature assessing the effectiveness of technology-based interventions for children and young people with T1DM on the diabetes self-management behaviours and prerequisites that are highlighted by current treatment guidelines as being crucial for effective diabetes management [2]. In this way, this review will identify aspects of national guidance which may be effectively promoted to children and young people using technological tools.

## Methods

Reporting of the systematic review followed the PRISMA (Preferred Reporting Items for Systematic reviews and Meta-Analyses) checklist [7].

### Eligibility

Peer-reviewed studies published in the English language prior to May 2017 were considered. Eligibility criteria were as follows:

- Participants: Aged between 2 and 18 years or described as ‘paediatric’, with a clinical diagnosis of T1DM (with or without comorbidities). For the purpose of this review this is also the definition of children and young people [8].
- Intervention: Interventions that supplement usual care with a health care strategy primarily delivered through a technology-based medium for the engagement of patients in self-management behaviours. Studies using continuous glucose monitoring (CGM) were included when participants were still required to conduct blood sugar checks and so they could be deemed to be purely an informational tool used to prompt self-management. Interventions targeting only parents/primary caregivers or other health stakeholders and not the child directly were excluded. Interventions which included a technology component as a secondary medium for delivery were excluded.
- Comparison: A comparison condition was not required for inclusion.
- Outcomes: Key behaviours (SMBG, insulin administration, physical activity and diet), prerequisites to behaviour (psychological supports) and indicators of behaviour (HbA1c) as highlighted by NICE guidance relating to diabetes self-management [2].
- Study design: Randomised or non-randomised studies and cohort studies. Single-case studies were excluded.

### Search methods

**Fig. 1:**
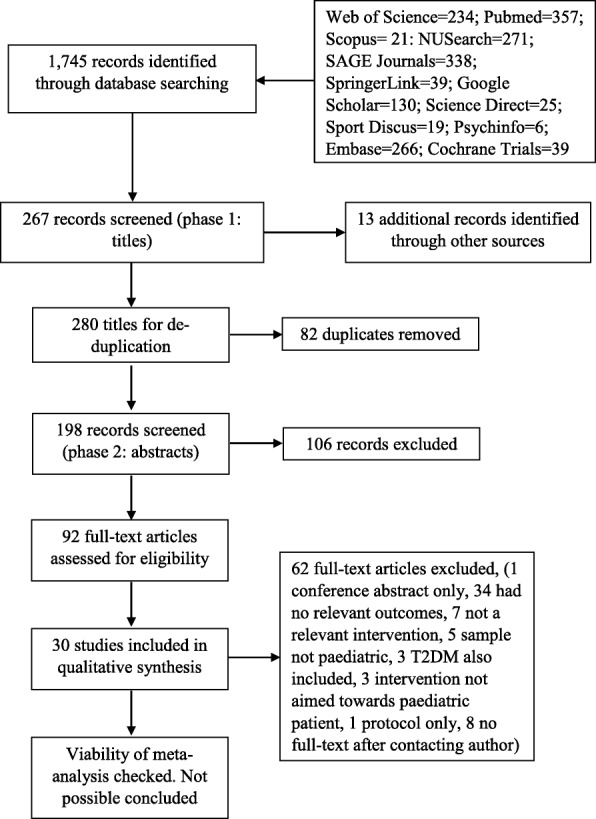
Search Process

Twelve databases were searched in April 2017 using the strategy shown in Fig. 1. Full literature search terms with their Boolean operators and the review protocol are available as supplementary materials (Supplementary Material 1 and 2). Titles were first screened for ineligibility (i.e. all articles were carried forward to abstract screening unless the title obviated exclusion). The reference lists of review articles were searched by hand. Following de-duplication, one reviewer (EK) screened abstracts of potentially relevant articles. If the abstract suggested potential eligibility, the full article was screened by EK and HQ independently. Reasons for exclusion were recorded with disagreements being resolved by a third reviewer (HB). Initial agreement between authors was 76% however, after discussion clarifying criteria, there was 100% agreement without need for the third reviewer.

### Data extraction and study quality

The full text of each included study was read critically by two reviewers independently (EK and HQ) and data were tabulated (EK). The following items were included: study details (author, publication year, aim, location, inclusion criteria), randomisation method (if applicable), study design, length of follow-up, comparison arm (if applicable), participant characteristics (gender, age, ethnicity etc.), intervention details (type, intensity etc.), recruitment and retention details (attrition rate, adherence etc.), outcomes and statistical analysis. Corresponding authors were contacted wherever insufficient detail was reported in the article.

### Measures of treatment effect

Reported data included effect sizes, 95% confidence intervals (CI) and *p* values, mean values and standard deviations (SD). A narrative synthesis approach was used to summarise the results [9]. Co-author and lead consultant at a paediatric diabetes clinic (TR) assessed clinical relevance of findings.

### Assessment of risk of bias

Evaluation of risk of bias in randomised controlled trials (RCTs) followed recommendations of the Cochrane Collaboration tool [10]. For non-randomised studies the Risk of Bias in Non-Randomised Studies of Interventions tool (ROBINS-I tool) was applied [11].

## Results

### Included studies

The database searches yielded 1745 records (see Fig. 1). After screening titles, 267 records remained. A further 13 records were identified following scrutiny of related articles. After de-duplication, 198 records remained. Abstract screening led to 106 articles being excluded, leaving 92 articles to be screened in full. Of these, 30 studies met the inclusion criteria for review. Details of included studies are provided in Table 1. Following selection, studies were grouped according to the behaviour, prerequisite or indicator measured; SMBG, insulin administration, dietary management (e.g. dietary habits, monitoring behaviours for diet, etc.), physical activity, psychological factors (e.g. self-efficacy) and HbA1c. Studies including more than one relevant outcome are included under more than one heading.

**Table 1:** Study characteristics

Study	Design	Medium	Setting	Age range	N	% Male	No. of patients lost to analysis	Intervention	Control group	Relevant outcomes	Adherence
Berndt et al. (2014) [30]	2 group RCT	Mobile phone app	Germany	8–18	68	39.7	0	Encouraged to use 3/day for 4 weeks	Usual care	HbA1c, self-efficacy	Below recommended amount
Boogerd et al. (2013) [31]	2 group RCT	Interactive online tool	Netherlands	11–21	62	35.5	12	Access for 9 months	Usual care	HbA1c, self-efficacy, knowledge	35–65% engagement with components
Clements & Staggs (2017) [21]	2 group non-RCT	Mobile phone app	USA	10–16	81	49.0	11	Access for at least 60 days	Usual care	SMBG	Synced 0.22 times a week
Dyal et al. (2017) [22]	Cohort	Interactive online tool	Canada	8–12	13	53.0	2	Access for 3 months, encouraged by avatars to log 3 times/day	None	SMBG	69% logged in the recommended amount
Franklin et al. (2006) [34]	3 group RCT	Text messaging support system	Scotland	8–18	126	53.8	1	Received 1–2 messages a day for 1 year	Usual care	HbA1c, self-efficacy, knowledge	Not relevant
Freeman et al. (2013) [41]	2 group RCT	Internet-based videoconferencing	USA	12–19	92	59.2	21	Up to 10 1–1.5 h sessions in a 12 week period	Usual care	Working alliance	Completed 7 sessions on average
Frøisland et al. (2012) [38]	Cohort	Mobile phone app	Norway	13–19	12	41.7	0	Access for 3 months, encouraged to use at least two 3 day periods	None	HbA1c	Not reported
Giani et al. (2016) [23]	Cohort	CGM	USA	8–17	61	52.0	0	Provided for 6 months	None	SMBG, insulin administration	Used average of 5.4 days a week at baseline, 3.4 days a week at 6 months
Goyal et al. (2017) [12]	2 group RCT	Mobile phone app	Canada	11–16	92	45.7	1	1 h tutorial at start to enable independent usage over 6 months	Usual care	SMBG, insulin administration	65% had low or very low engagement
Han et al. (2015) [29]	3 group RCT	Text messaging	USA	10–17	30	43.0	0	Received 1 text message a day for 26 weeks	Usual care	HbA1c	2/4 educators accessed the program
Henkemans et al. (2017) [42]	3 group RCT	Interactive robot	Netherlands	7–12	28	48.1	1	Played a quiz with robot during 3 consecutive clinic appointments	Usual care	Knowledge, need satisfaction	Only 100% completers included
Harris et al. (2015) [16]	2 group RCT	Internet-based videoconferencing	USA	12–19	90^c^	55.0	24	Up to 10 1–1.5 h sessions in a 12 week period	Face-to-face skills sessions	SMBG	Completed 5.8 sessions on average
Herbert et al. (2016) [24]	Cohort	2-way text messaging software	USA	13–17	23	39.0	0	Daily interactive prompts and educational text messages for 6 weeks	None	SMBG	78% of text messages responded to
Kowalska et al. (2017) [28]	2 group RCT	Computer software with automatised food and insulin calculation	Poland	< 18	106	39.6	2	Encouraged to use for at least 50% of meals for 26 weeks	Usual care	HbA1c	41.5% used the recommended amount
Landau et al. (2012) [17]	2 group RCT	Internet-based glucose monitoring system	Israel	11–20	70	15.0	3	Maximum of weekly calls for 6 months	Usual care	SMBG	66.6% accessed the recommended amount
Lehmkuhl et al. (2010) [19]	2 group RCT	Telehealth behavioural therapy	USA	9–17	32^3^	28.1	0	3 × 15 min sessions a week for 12 weeks	Usual care	SMBG	All sessions completed
Mulvaney et al. (2010) [32]	2 group RCT	Internet-based program	USA	13–17	72	55.6	0	6 multimedia stories over 11 weeks	Usual care	HbA1c, problem solving	63–76% engaged with various components
Mulvaney et al. (2012) [26]	Cohort	Mobile phone-based ecological momentary assessment	USA	12–17	50	50.1	0	2 calls a day for 10 days	None	SMBG, insulin administration	59.4% total call records with complete data
Mulvaney et al. (2012b) [39]	2 group non-RCT	Personalised automated text messaging	USA	13–17	28	57.0	5	10 text messages a week for 3 months	Usual care	HbA1c	2.9 messages a week responded to
Newton et al. (2009) [27]	2 group RCT	Text messaging	New Zealand	11–18	78	47.0	0	Weekly text messages/ pedometer reminders over 12 weeks	Usual care	Physical activity	All messages sent
Nordfeldt et al. (2003) [37]/ Nordfeldt et al. (2005) [20]	3 group RCT	Personalised video tapes	Sweden	2–18	332	83.0	0	2 mailed videotapes	Usual care	SMBG, HbA1c	Use range between 1 and 20 times
Nunn et al. (2006) [35]	2 group RCT	Telephone support and educational program	Australia	3–16	139	56.0	16	Bimonthly 15–30 min telephone calls for 7 months	Usual care	HbA1c, knowledge	Not stated
Pinsker et al. (2011) [40]	Cohort	Website	USA	Omitted^a^	52	51.9	20	Given access for 6 months	Non-users	HbA1c	Logged in 4 or more times during the study
Rachmiel et al. (2015) [25]	2 group non-RCT	CGM	Israel	1–17	149	47.7	0	Provided with for 1 year	Usual care	SMBG	38% used 75% of the time, 50% stopped using by 6 months and 66% by 1 year
Raiff et al. (2016) [13]	2 group RCT	Internet-based program	USA	13–18	52	58.5	11	Given access for 20 days	Non-users	SMBG	All participants used on at least 10 days
Rami et al. (2006) [36]	2 group crossover RCT	Mobile phone-based support program	Austria	10–19	36	55.6	0	Received 1 text a week for 3 months and access for 6 months	Usual care	HbA1c	25% engaged < 50% of the recommended amount
Schiaffini et al. (2016) [14]	2 group RCT	Website	Italy	Omitted^b^	29	37.9	2	Access for 5 years, monthly reminders to access	Usual care	SMBG, insulin administration	2 patients disengaged in the fifth year
Whittemore et al. (2010) [33]	2 group RCT	Interactive internet sessions	USA	13–16	12	42.0	0	1 session a week for 5 weeks	Internet education intervention	HbA1c, self-efficacy	83% completed all sessions
Whittemore et al. (2012) [18]	2 group RCT	Interactive internet sessions	USA	11–14	320	45.0	0	1 session a week for 5 weeks	Internet education intervention	SMBG, self-efficacy	78% completed at least 4 sessions
Whittemore et al. (2016) [15]	2 group RCT	Internet psychoeducational program	USA	11–14	124	37.4	0	Prompted to login 2 times a week for 4 weeks	Open access website	SMBG, self-efficacy	85% logged in at least once overall

^a^Age range not stated. Described as ‘children’, mean age of 11.2. ^b^Age range not stated. Recruited from children’s hospital, mean age of 13. ^c^N refers to child-parent dyads, only child data considered in this review

#### Interventions reporting on frequency of SMBG

Nine RCTs [12–20] and six non-RCTs [21–26] targeted SMBG as an outcome. Full quality assessment details are provided in the Tables. For the RCTs (Table 1), six studies demonstrated low risk of bias [13, 15, 17–20], two demonstrated moderate risk [12, 16] and for one the level of risk was unclear [14]. For the non-RCTs (Table 2), two demonstrated low risk of bias [23, 25], one was low-moderate risk [24], one was moderate risk [21] and two were high risk [22, 26].

**Table 2:** Summary of clinical significance of findings relating to each main outcome of interest

Study	Clinical significance of findings	Clinical relevance
Self-monitoring of blood glucose (SMBG)		
Clements & Staggs (2017) [21]	Positive link found but 1/3 did not use app so it is likely that individuals who often SMBG use the app rather than the app encouraging more SMBG. Factor of increase (2.3) reported but exact figures not given so difficult to interpret i.e. could be increase from 1 to 2.3 which would impact very little or 3 to 6.9 which would impact a great deal	Likely low
Harris et al. (2015) [16]	25-item measure but does not give any measure of frequency and so impossible to say	Unclear
Mulvaney et al. (2012) [26]	1/3 of the cohort sent through no data, percentage of missed blood glucose tests reported but actual frequency of SMBG not given and there is very high variability between the groups i.e. some participants missed very few and some missed almost all tests thus for some findings could be clinically meaningful but not for others	Unclear
Rachmiel et al. (2015) [25]	Difference between continuous and intermittent users expected to have clinical significancethough would also lead to concomitant increase in discomfort from testing	Likely high
Raiff et al. (2016) [13]	An increase of 2 to 4 tests per day would be expected to result in clinical improvements	Likely some
Frequency of SMBG and insulin administration behaviour		
Giani et al. (2016) [23]	Improvements found amongst those who monitored regularly at the beginning of the study but not those who initially monitored poorly. This suggests the intervention was effective but only for those who did not need it	Likely low
Schiaffini et al. (2016) [14]	Can be assumed that the intervention increased SMBG and insulin administration to a degree that would be clinically significant. Unclear whether this is due to the electronic platform or feedback from the clinical team	Likely some
HbA1c		
Mulvaney et al. (2012b) [39]	Nature of control group (matched historical control) is inappropriate to enable estimation of clinical significance	Unclear
Pinsker et al. (2011) [40]	No statistical significance found but demographic characteristics of the two groups are not provided and so it is not clear if groups were matched	None
Rami et al. (2006) [36]	No statistical significance reported	None
HbA1c and self-efficacy		
Franklin et al. (2006) [34]	Improvements only reported in the intensive insulin group suggesting that the technology-based intervention was not primarily responsible for differences	None
Self-efficacy		
Berndt et al. (2014) [30]	Measure likely taken immediately after receiving information from the clinical team and so probably does not reflect real changes in self-efficacy	Likely low
Whittemore et al. (2012) [18]	Intervention appears to have been no more effective than control	Likely low
Need satisfaction (SDT)		
Henkemans et al. (2017) [42]	Unclear how findings relate to clinical outcomes	Unclear

##### Study characteristics

Sample sizes ranged from 13 to 332 participants. The proportion of male participants ranged from 15 to 83%. All samples were recruited from paediatric clinics within one or more hospitals. The age of participants from all but one study ranged from two to 18 years. One study included participants aged 12–19 though participants were receiving treatment from a paediatric clinic and described as youth.

Interventions included text messaging [*n* = 2 [24, 26]], mobile phone applications [*n* = 2 [12, 21]], telephone-delivered behavioural therapy [*n* = 1 [19]], video tapes [*n* = 1 [20]], teleconferencing [*n* = 1 [16]], websites [*n* = 1 [13]], CGMs [*n* = 2 [23, 25]] and interactive online tools [*n* = 5 [14, 15, 17, 18, 22]]. None of the studies mentioned a theoretical basis to the intervention.

Eleven of the studies employed a measurement of SMBG frequency via glucometer download (*n* = 6), direct daily telephone report (*n* = 1), retrospectively self-reported questionnaire (*n* = 1), video upload (*n* = 1) or daily website logs (*n* = 2). Four studies employed questionnaires to obtain a rating of SMBG (Diabetes Self-Management Profile, the Self-Care Inventory, 52-item Self-Management of Type 1 Diabetes in Adolescence Scale).

Only 3/9 RCTs reported greater SMBG frequency in the intervention group [13, 14, 16]. One RCT found non-significant trends towards higher SMBG following intervention [20]. One RCT reported no change in SMBG [18] and another reported no difference relative to a control group [15] following intervention. Two RCTs found no change in SMBG following intervention even when intervention engagement was considered [12, 17]. One RCT found improved SMBG after intervention but no improvement relative to a control group [19].

Raiff and colleagues [13] reported an increase of 1.3 SMBG tests per day in participants receiving motivational interviewing and non-contingent rewards via a webcam as opposed to face-to-face (*p* < 0.01). This increased this to 1.96 tests per day (*p* < 0.01) when contingent rewards were offered. Schiaffini and colleagues [14] reported an increase in SMBG tests per day (5.5 ± 0.7 vs. 3.8 ± 0.7; *p* = 0.00) after 5 years, in a group receiving monthly tele-contacts from their clinical team relative to usual care alone. Harris and colleagues [16] reported main effects of intervention from baseline to 12 weeks on adherence score following ten sessions of behavioural family systems therapy delivered via Skype (47.56 ± 12.78 to 53.22 ± 12.64, *d* = 0.45, *p* < 0.001) and 6 months (47.56 ± 12.78 to 50.94 ± 12.38, *d* = 0.18, *p* < 0.001). Nordfeldt and colleagues [20] found no changes in SMBG frequency 24 months after participants received either personalised video tapes and a brochure (2.7 ± 1.7 vs. 3.0 ± 1.5 times/day, *p* = n.s.), generalised videos (2.4 ± 1.5 vs. 2.8 ± 2.2) or standard care exhibited alone (2.6 ± 1.5, 2.8 ± 1.7, *p* = n.s.). Similarly, Goyal and colleagues [12] found no difference in SMBG between a group given access to a mobile phone application incentivising SMBG (6.33 ± 0.45) and a control group (6.24 ± 0.57), after 1 year (*p* = 0.90). Landau and colleagues [17] recorded SMBG frequency via monthly glucometer downloads and found that download frequency was similar every month for 6 months (specific values not given) following an internet-based blood glucose monitoring system. Two studies reported no difference in self-reported SMBG between an internet coping skills intervention and control after 6 months [31.8 ± 5.6 vs. 32.6 ± 5.9, *p* = 0.02 [14]], [− 0.02 ± 0.03, *t* = 0.49 vs. -0.00 ± 0.03, *t* = 0.97, *p* = 0.65 [17]]. One study found an improvement in self-reported SMBG over time (42.3 to 48.4; *F*(1,27) = 16.3, *p* < 0.01) but no intervention effect at 3 months after 36 behavioural therapy sessions delivered via telephone [19].

Of the six non-RCTs, one found a non-significant trend towards higher SMBG following intervention [22]. Four studies found greater SMBG frequency to be associated with higher intervention use [21, 23, 25, 26]. One study found no change in SMBG following intervention at any measurement time-point even when intervention engagement was considered [24].

Herbert and colleagues [24] found a trend towards lower daily SMBG frequency after 6 weeks of receiving daily educational text messages prompting SMBG (*t*(16) = 17.3, *p* = 0.10). Mulvaney and colleagues [26] reported significantly fewer missed blood glucose tests in a group which adhered adequately to wearing a CGM than a group which did not (16.81% vs. 36.50%; Mann-Whitney *U* = 10.0, *Z*-statistic = 4.65, *p* < .001). Likewise, Rachmiel and colleagues [25] and Giani and colleagues [23] found that participants who consistently used their CGM exhibited a higher frequency of daily SMBG than those who did not after 1 year (10.6 ± 4.9 vs. 6.3 ± 2.8, *p* = 0.01) and 6 months (> 8 times/day vs. < 8 times/day, *p* = 0.05), respectively. Clements and Staggs [21] also found participants increased their SMBG frequency by 2.3 each time they synchronised data to a mobile phone application (*p* < 0.01, *CI* = 1.82, 2.90). Dyal and colleagues [22] did not perform a statistical analysis of data but reported daily logs of SMBG to increase from < 3 a day at baseline to as many as 34 logs a day after 5 weeks of accessing an online interactive tool.

#### Interventions reporting on insulin administration behaviour

Two RCTs [12, 14] and two non-RCTs [23, 26] included insulin administration behaviour as an outcome. Full quality assessment details are provided in the Tables. One RCT demonstrated moderate risk of bias [12] and one had unknown risk [14] (Table 1). Of the two non-RCTs, one demonstrated low risk of bias [23] and another demonstrated high risk of bias [26].

##### Study characteristics

Sample sizes ranged from 29 to 92 participants. The proportion of male participants ranged from 37.9 to 52%. All samples were recruited from paediatric clinics at one or more hospitals. The age of participants from three studies ranged from eight to 17 years. One study did not state the age range of participants but described the sample as ‘paediatric’ and mean age was 13 years.

Interventions included CGM [*n* = 1 [23]], a mobile phone app [*n* = 1 [12]], an automated interactive telephone response system [*n* = 1 [26]] and a website [*n* = 1 [14]]. All of these studies also measured SMBG as an outcome.

Two studies reported the proportion of insulin doses missed via meter download and self-report. One of these also reported the proportion of incorrectly administered doses, one study used self-reported self-initiated adjustments to the insulin regimen and one study used self-reported daily records of insulin boluses per day.

One RCT found improvements to insulin administration following intervention [14]. The other RCT found no effect of intervention on the number of self-initiated adjustments to the insulin regimen relative to a control [12].

Schiaffini and colleagues [14] provided participants with a standardised educational programme encouraging better self-monitoring. Half of the participants also received access to an online website and were encouraged to access it monthly for personalised feedback. After five years, intervention participants reported administering more insulin boluses per day relative to baseline (4.2 ± 1.0 vs 3.3 ± 1.0, *p* = 0.03). In the study by Goyal and colleagues [12], participants receiving access to a mobile self-monitoring application reported 1.85 ± 2.3 participant-initiated adjustments to the insulin dose at baseline and 1.77 ± 2.7 after 12 months. Participants without access to the app reported 2.08 ± 3.4 self-initiated adjustments at baseline and 1.10 ± 1.3 at 12 months; these differences were not significant (*p* = 0.25).

Of the non-RCTs, one study found improvements to insulin administration following intervention [23]. The other non-RCT found no association of adherence to intervention with insulin administration [26].

Giani and colleagues [23] reported that participants who were provided with a CGM for 6 months and used it on average 6–7 days/week during that period, were significantly less likely to report missing insulin doses (18%) than those using it 0–5 days/week (47%, *p* = 0.02). Mulvaney and colleagues [26] found that individuals with good adherence to a mobile-phone based ecological momentary assessment intervention (real time sampling of behaviours/experiences) reported similar missed (*r*_*s*_ = 0.14, *p* = 0.92) and correct (*r*_*s*_ = 0.09, *p* = 0.54) insulin doses to those with low adherence.

#### Interventions reporting on dietary management behaviour

No studies included dietary management behaviour as an outcome.

#### Interventions reporting on physical activity behaviour

One of the included studies targeted physical activity as an outcome [27]. This study demonstrated moderate risk of bias (Table 1).

##### Study characteristics

This study used weekly text messaging alongside provision of an open pedometer to encourage participants to reach 10,000 steps a day over 12 weeks (*n* = 78; 47% male). Participants aged between 11 to 18 years were recruited from four adolescent diabetes outpatient services in New Zealand. Measures included pedometer step count, and self-reported physical activity using the New Zealand Physical Activity Questionnaire and SPARC-Long Physical Activity Questionnaire.

##### Effect of intervention on physical activity levels

The study found a non-significant trend towards decreased daily step count in both intervention (*CI* = 1407 to 1364) and control groups (*CI* = 1947 to 266, *p* = 0.4), following 12 weeks of intervention. A non-significant trend towards increased self-reported physical activity was evident in both groups (intervention: + 48.4 min/week, control: + 38.5 min/week more, *p* = 0.9).

#### Interventions reporting on glycaemic control (HbA1c)

A total of ten RCTs [28–37] and three non-RCTs [38–40] measured HbA1c as a clinical indicator of diabetes self-management. Five of the RCTs demonstrated low risk [28, 33–35, 37] three RCTs had unknown risk [29, 30, 36], one RCT had moderate risk [32] and one had moderate-high risk of bias [31]. Of the non-RCTs, two demonstrated a moderate risk of bias [38, 40] and one demonstrated a high risk of bias [39].

##### Study characteristics

Sample sizes ranged from 12 to 332 participants. The proportion of male participants ranged from 35.5 to 57%. All samples were recruited from paediatric clinics at one or more hospitals. The age of participants ranged from 3 to 21 years. All participants were receiving treatment from a paediatric clinic and/or described as adolescents or children.

Interventions included text messaging [*n* = 3 [29, 34, 36]], mobile phone applications [*n* = 3 [30, 38, 39]] telephone-delivered programmes [*n* = 1 [35]], video tapes [*n* = 1 [37]], websites [*n* = 1 [40]], interactive online tools [*n* = 3 [31–33]] and USB kitchen scales [*n* = 1 [28]].

Two RCTs found positive intervention effects on HbA1c [34, 36]. Seven RCTs found no effect of intervention on glycaemic control [28–32, 35, 37]. One study provided only descriptive statistics and did not conduct statistical analyses of these data [33].

Franklin and colleagues [34] reported a significant improvement in HbA1c following 12 months of text messaging support (1–2 messages per day) and intensive insulin therapy relative to a control group (9.2 ± 2.2, *95%CI* = − 1.9, − 0.5, *p* < 0.001). Similarly, Rami and colleagues [36] used a randomised crossover design to examine the efficacy of a telemedical support system encouraging SMBG. HbA1c significantly improved in the intervention group (intervention first group: 9.05% [8–11.3%] at baseline, 8.9% [6.9–11.3%] at 3 months, and 9.2% [7.4–12.6%] at end, intervention second group: 8.9% [8.3–11.6%], 9.9% [8.1–11%], and 8.85% [7.3–11.7%], *p* < 0.05). Conversely, Nordfeldt and colleagues [37] did not find any differences in change in HbA1c between groups receiving personalised video tapes, generalised video tapes or traditional care (values not reported). Similarly, Berndt and colleagues [30] found no difference in improvements in HbA1c between groups receiving four weeks of access to a mobile phone application for 24-h self-monitoring and feedback, relative to a control group (8.96% ± 2.23 to 7.99% ± 1.26 vs. 8.84 ± 1.71 to 8.12% ± 1.1 *p* = n.s.). Kowalska and colleagues [28] provided patients with a nutritional database and a USB kitchen scale for 26 weeks. Relative to a control group the intervention group showed no change in HbA1c over time after 3 months (7.2 ± 1.1 vs. 7.6 ± 1.1, *p* = 0.09) or 6 months (7.4 ± 1.2 vs. 7.6 ± 0.8, *p* = 0.16). Nunn and colleagues [35] found no difference in the change in HbA1c between a group receiving bimonthly telephone support delivering an educational programme and a usual care group (8.15% ± 1.14 to 8.85% ± 1.29 vs. 8.32% ± 1.01 to 8.82% ± 1.10, *p* = 0.24). Similarly, Mulvaney et al., [32] found no difference in change over time between a group receiving six multimedia stories over 11 weeks and prompts to complete problem-solving tasks, and a usual care group (9.1% ± 1.9 to 9.1% ± 1.8 vs. 8.2 ± 1.2 to 8.5% ± 1.3, *p* = 0.27). Han and colleagues [29] reported no difference in HbA1c change in groups receiving symptom-related text messages (− 0.37, *d* = 0.39, *p* = 0.12), symptom and knowledge-related text messages (− 0.03, *d* = 0.04, *p* = 0.46), and standard care alone (− 0.21, *d* = 0.47, *p* = 0.87). Boogerd et al., [31] reported no differences in change over time between a group receiving nine months of access to an online interactive intervention and a group receiving usual care (*F*[1,61] = 0.16, *p* = 0.69). Whittemore and colleagues [33] reported a trend towards maintaining stable HbA1c at three and six months in a group receiving an internet coping skills program. Alternatively, a control group showed a trend towards increasing HbA1c. Statistical analysis of this outcome was not conducted.

One non-RCT found positive intervention effects on HbA1c [39]. One non-RCT found no effects [38] and one found positive associations with HbA1c but only when engagement with the intervention was considered [40].

Mulvaney et al., [39] found a significant interaction between group and time in a study comparing patients receiving ten text messages per week for three months and a matched historical control group (8.8% ± 2.1 to 8.8% ± 2.1 vs. 9.9% ± 2.3 to 8.92% ± 2.2, *p* = 0.01). Similarly, Pinsker and colleagues [40] found a significant improvement in HbA1c in patients using an online portal and educational materials relative to a group who did not utilise their access to the portal (users: 10.5 to 9.1%; non-users: 9.5 to 10.4%, *p* = 0.03). Conversely, Frøisland and colleagues [38] found no changes in HbA1c after giving children and young people access to a mobile phone-based diabetes picture diary which linked automatically to their glucometer (8.3% vs. 8.1%, *p* = 0.38).

#### Interventions reporting on psychosocial or cognitive indicators of improved self-management

A total of ten RCTs measured psychosocial or cognitive indicators of self-management [15, 18, 30–35, 41, 42] Five of these studies demonstrated low risk of bias [15, 18, 33–35] one demonstrated moderate risk [32], one study demonstrated moderate-high risk [31] and three RCTs had unknown risk of bias [30, 41, 42].

##### Study characteristics

Sample sizes ranged from 12 to 320 participants. The proportion of male participants ranged from 35.5 to 57%. All samples were recruited from paediatric clinics at one or more hospitals. The age of participants ranged from three to 21 years. Two of the studies included participants aged over 18 years. Both were receiving treatment from a paediatric clinic and/or described as adolescents.

Interventions included mobile phone applications [*n* = 1 [30]], telephone call support [*n* = 1 [35]], videoconferencing [*n* = 1 [41]], text messaging [*n* = 2 [32, 34]], internet-based programs [*n* = 4 [15, 18, 31, 33]] and personal robots [*n* = 1 [42]].

Six studies measured self-efficacy for diabetes management using the Diabetes Self-Efficacy Scale, the Self-Efficacy for Diabetes Scale, the Self-Efficacy for Diabetes Management Measure or the Confidence in Diabetes Self-Care Questionnaire. One study employed a measure of self-determination constructs via the Basic Need Satisfaction in Relationships Scale. One study used the Working Alliance Inventory to provide a measure of the relationship between the child and their clinical team. Four studies measured change in diabetes knowledge using the Diabetes Knowledge Questionnaire, Diabetes Knowledge Score, Test of Diabetes Knowledge and a bespoke 30-question measure. One study measured problem solving ability via the Diabetes Problem Solving Behaviours Scale.

Four studies found improvements in at least one of the psychological or cognitive parameters measured in their studies following intervention [18, 30, 34, 42]. Alternatively, six studies found no improvements after receiving an intervention [15, 31–33, 35, 41].

Berndt and colleagues [30] found a significant improvement in self-efficacy in a group of young patients encouraged to use “Mobil Diab”, a mobile diabetes management system, three times a day for four weeks (7.54 ± 0.85 to 8.04 ± 1.22, *p* = 0.04). A comparison group receiving usual care did not significantly improve their self-efficacy (7.22 ± 1.64 to 7.65 ± 1.24, *p* = 0.12). In a study by Henkemans and colleagues [42] participants played games with a neutral robot or a personal robot during three consecutive clinic visits. Following the third session, participants playing with the personal robot demonstrated higher self-reported need satisfaction (*Z* = 2.33, *p* = 0.02). Participants playing with the robot also answered significantly more questions correctly than those who did not play the quiz (*F* [1, 45]=7.27, *p* = 0.00). Franklin and colleagues [34] reported greater improvements in self-efficacy (*95%CI*: 2.6, 7.5, *p* = 0.00) in a group receiving 1–2 text messages a day via “Sweet Talk” than a group receiving usual care, but not in diabetes knowledge (*95%CI*: -1.5, 1.4, *p* = 0.3). Whittemore and colleagues [18] reported a significant increase in self-efficacy of children and young people receiving five weekly online cognitive skills sessions (TEENCOPE) and another group given access to a generic diabetes website (*p* < 0.001). Other studies of TEENCOPE found no better outcomes than alternative treatments. Whittemore and colleagues [33] found non-significant trends towards improved self-efficacy after five weekly cognitive skills sessions delivered online (55.5 to 39.0, *p* = 0.20). Whittemore and colleagues [15] later conducted another study, which combined the earlier developed TEENCOPE with an online problem solving program and found no effect of intervention or a generic website on self-efficacy (*SE* = − 1.20 ± 0.84 vs. -0.43 ± 0.85, *p* = 0.52). Boogerd and colleagues [31] reported no difference in change over time for self-efficacy (*F*[1,61] = 2.55, *p* = 0.12) or diabetes knowledge (*F*[1,61] = 0.09, *p* = 0.77) between a group receiving a nine-month online interactive treatment called Sugarsquare, relative to a group receiving regular emails. Freeman and colleagues [41] found no difference in working alliance (221.62 vs. 224.84, *p* = 0.53) following ten behavioural health care sessions delivered via Skype or in clinic. Mulvaney and colleagues [32] found six tailored multimedia stories over 11 weeks to have no differential effect on problem solving ability relative to usual care alone (3.5 ± 0.5 to 3.6 ± 0.5 vs. 3.4 ± 0.6 to 3.3 ± 0.7, *p* = 0.23). Nunn and colleagues [35] reported no difference in change in diabetes knowledge following 7 months of bimonthly supportive telephone discussions or continuing usual care (79.1 ± 57.4 to 84.5 ± 52.7 vs. 77.5 ± 55.7 to 82.4 ± 55.6, *p* = 0.34).

## Discussion

This review demonstrates that technology-based health interventions can exert minor positive influences on SMBG (as a behavioural outcome), psychological or cognitive outcomes, and clinical indicators of diabetes self-management (e.g. HbA1c). Physical activity and dietary practices are rarely targeted by technology-based interventions and almost never measured as outcomes, despite evidence from non-technology-based interventions of positive effects on the management of T1DM in children and young people [43, 44]. Technology-based interventions that target physical activity and dietary behaviours in studies that measure these factors as outcomes, are therefore encouraged.

The most positive finding was that the majority of studies exploring SMBG behaviour found that children and young people monitored their blood glucose more frequently following exposure to technology-based interventions. This is important since greater self-monitoring frequency is associated with better diabetes management [45]. However, only three of these studies produced results that were deemed to be clinically relevant (Table 1). In the case of two of these studies this may be explained by the longer follow-up period used relative to that in other included studies [14, 25]. The third study with positive findings for SMBG only recruited patients who were not adherent to their existing self-management routine, and so may have had greater potential for demonstrating outcome improvement [13]. Future studies should therefore test interventions over a period of at least one year and may wish to target non-adherers.

Psychosocial and cognitive supports have also been associated with better diabetes outcomes [46–48]. Approximately half of the studies including measures of glycaemic control and/or cognitive or psychosocial parameters reported positive intervention effects. Positive changes were most common for self-efficacy. This is an important finding as self-efficacy has previously been identified as one of the most important predictors of disease management in children with chronic disease [49]. Future research in this area may seek to include technology-based interventions that aim to build self-efficacy specifically. However, none of the findings in the included studies were deemed to be clinically significant. The articles failing to report positive outcomes tended to involve a smaller study sample and scored lower on study quality, and as such they may have lacked power or sensitivity to detect changes. There is a need for high-quality research evidence with more complete descriptions of blinding procedures, outcome reporting and usual care, in order to fully assess the impact of technology-based interventions on glycaemic control and psychosocial/cognitive outcomes.

None of the studies found usual care alone to produce better outcomes than intervention conditions. While usual care must always be provided where possible, technology interventions could be useful in instances when the delivery of usual care is interrupted beyond the control of healthcare practitioners. For instance, if a patient frequently misses clinic appointments or is planning a trip away from their regular clinical care team, then technological interventions may be helpful in maintaining the delivery of key processes, such as HbA1c monitoring, provision of key information, goal setting etc., which might otherwise be missed. This could also apply to common transitional events during which usual care could be interrupted, such as family moves to new catchment areas (for schools and hospitals) and in later years the young person moving away to college or university [50]. Further, given evidence reported in a previous study by Petry et al. [51] that monetary reinforcers encourage greater SMBG of adolescents over a 12-week period, technology could be investigated as a tool for increasing longevity of reward effects, through facilitating delivery of rewards or even replacing monetary rewards with digital ones, given adolescents affinity for technological devices. This review suggests some promise for the use of technology-based interventions to promote aspects of the NICE guidance, but urges caution as evidence of efficacy is currently equivocal.

For the majority of included outcomes, clinical relevance was deemed to be little or none. Three studies demonstrated some to high clinical relevance for SMBG [13, 14, 25]. Many of the studies had methodological weaknesses or were poorly reported. A number of studies were ostensibly looking at the frequency of self-monitoring of blood glucose (SMBG) but did not give information on the actual number of blood glucose tests performed, or they relied on self-reporting of adherence to SMBG, which is well recognised as not always being reliable in young people with diabetes. Where HbA1C was used as an outcome measure, there was generally no difference between intervention and control groups or inappropriate control groups were used. In those studies where clinically meaningful outcomes were reported there was also significant direct personal intervention from the study team. This makes it very difficult to determine whether it was the technology or the additional contact from the study team which led to any improvements. Full details regarding clinical relevance of findings are provided in Table 1.

The majority of included studies were RCTs all of which were randomised at participant level. It is therefore impossible to estimate contamination bias of studies though this is likely to be low as interventions were predominantly delivered via personal devices. Participant characteristics in the control and intervention conditions at baseline were similar in most studies, however the variables measured varied in depth and type. Where found, differences were minor and were statistically controlled in comparative analysis, but the risk of subsequent bias in intervention outcomes remains. Just under half of included studies reported employing an intention-to-treat analysis, the remainder may have employed this type of analysis but failed to report it. It is therefore unclear to what extent selection bias has influenced findings.

The inclusion of non-RCTs increased the depth of the review but also the potential for researcher bias and this may have obscured interpretation of effectiveness.

Level of contact with the technology under investigation varied greatly between interventions, from multiple times per day to single follow-up time points. It is therefore challenging to attribute the success of a particular intervention to a specific intervention component. More than half of the studies assessed outcomes over the medium term (six months to one year). Although attrition appeared to be higher in studies involving a potentially greater burden of time or scheduled interactivity for the participant (e.g. videoconferencing), attrition across the studies was generally low. This supports previous findings that technology based interventions are generally well-accepted by children and young people with T1DM [4].

One focus of future research could be to increase the emphasis on the behaviour change aspect of technology-based health interventions for children and young people with T1DM. Evidence of efficacy tended to be stronger when adherence to the intervention was considered. However, few studies explicitly framed their interventions in behaviour change theory (or reported doing so), though it is possible that theoretical components were used in the development of messages. Future studies should ensure that interventions are developed and framed in the context of current behaviour change theory and that this aspect is well reported.

There is a need to examine technologies aiming to promote healthy lifestyle behaviours that are advocated in NICE guidelines and known to influence diabetes management outcomes. For example, physical activity is a beneficial health behaviour for all young people generally and for those with T1DM specifically due to its role in promoting insulin efficiency and glycaemic control. Children and young people with T1DM tend to fall short of physical activity guidelines and may be less active than their peers without T1DM [52]. There is some existing evidence demonstrating the potential feasibility and acceptability of technology-based studies on physical activity behaviours in paediatric diabetic populations [4] although this needs to be further investigated.

Only articles published in the English language were included; thus, other relevant studies in other languages may have been missed. Due to publication bias it is also possible that relevant findings were missed [53]. As no studies measuring dietary management behaviours fitted our inclusion criteria, and very few studies measured physical activity or insulin administration, meaningful conclusions regarding these outcomes are precluded. The present review included a wide age range to be as inclusive as possible, however, we acknowledge that the outcomes of interventions will be influenced by age. Finally, it was the intention of this review to take a behavioural standpoint. Therefore, the focus here is on the *self-management behaviours* and prerequisites most likely to be targeted by technology-based interventions and measured as outcomes, as opposed to focusing on the *types* of technologies utilised. Whilst this may be a limitation of this review, other systematic reviews have provided this perspective. This review offers a novel approach to the use of technology to advocate the behaviours and prerequisites specified in the current NICE guidance for the self-management of paediatric T1DM.

## Conclusions

NICE guidance proposes a number of key behaviours [2], prerequisites and predictors to the effective management of T1DM. Published evidence around the use of technology-based interventions has neglected to include or report on outcomes pertaining to some of these (e.g. diet, physical activity and insulin administration). We identified a number of efficacious studies indicating promise for the use of technology as a platform to deliver self-management interventions, specifically for SMBG and building self-efficacy. Further, technology-based interventions performed no worse than usual care alone in all studies and attrition was generally low indicating patient acceptability. As such, we conclude that technology-based interventions may have merit for promoting some of the guideline objectives for the management of T1DM in children and young people. Further work is needed to ascertain which elements of interventions are most likely to produce clinically relevant outcomes.

## Abbreviations

CGM: Continuous glucose monitoring
NICE: National Institute for Health and Care Excellence
NIHR: National Institute for Health Research
PRISMA: Preferred Reporting Items for Systematic reviews and Meta-Analyses
RCT: Randomised controlled trial
RfPB: Research for Patient Benefit
ROBINS-I tool: Risk of Bias in Non-Randomised Studies of Interventions tool
SD: Standard deviation
SMBG: Self-monitoring of blood glucose
T1DM: Type one diabetes mellitus
